# Changes in cardiac output with hemodialysis relate to net volume balance and to inferior vena cava ultrasound collapsibility in critically ill patients

**DOI:** 10.1080/0886022X.2020.1726384

**Published:** 2020-02-12

**Authors:** Matthew J. Kaptein, John S. Kaptein, Christopher D. Nguyen, Zayar Oo, Phyu Phyu Thwe, Myint Bo Thu, Elaine M. Kaptein

**Affiliations:** a Department of Medicine, Division of Nephrology, Loma Linda University Medical Center, Loma Linda, CA, USA; b Department of Medicine, Division of Nephrology, University of Southern California, Los Angeles, CA, USA

**Keywords:** Intravascular volume, ultrafiltration, renal replacement therapy, intradialytic hypotension, Cardiac output, inferior vena cava ultrasound

## Abstract

Cardiac output may increase after volume administration with relative intravascular volume depletion, or after ultrafiltration (UF) with relative intravascular volume overload. Assessing relative intravascular volume using respiratory/ventilatory changes in inferior vena cava (IVC) diameters may guide volume management to optimize cardiac output in critically ill patients requiring hemodialysis (HD) and/or UF.

We retrospectively studied 22 critically ill patients having relative intravascular volume assessed by IVC Collapsibility Index (IVC CI) = (IVCmax-IVCmin)/IVCmax*100%, within 24 h of cardiac output measurement, during 37 intermittent and 21 continuous HD encounters. Cardiac output increase >10% was considered significant. Net volume changes between cardiac outputs were estimated from “isonatremic volume equivalent” (0.9% saline) gains and losses.

Cardiac output increased >10% in 15 of 42 encounters with IVC CI <20% after net volume removal, and in 1 of 16 encounters with IVC CI ≥20% after net volume administration (*p* = 0.0136). All intermittent and continuous HD encounters resulted in intradialytic hypotension. Net volume changes between cardiac output measurements were significantly less (median +1.0 mL/kg) with intractable hypotension or vasopressor initiation, and net volume removal was larger (median −22.9 mL/kg) with less severe intradialytic hypotension (*p* < 0.001). Cardiac output increased >10% more frequently with least severe intradialytic hypotension and decreased with most severe intradialytic hypotension (*p* = 0.047).

In summary, cardiac output may increase with net volume removal by ultrafiltration in some critically ill patients with relative intravascular volume overload assessed by IVC collapsibility. Severe intradialytic hypotension may limit volume removal with ultrafiltration, rather than larger volume removal causing severe intradialytic hypotension.

## Introduction

The goal of volume assessment and management in critically ill patients is to optimize relative intravascular volume in an attempt to improve cardiac output (CO) and tissue perfusion [1,2]. Reliable assessment of relative intravascular volume is essential for appropriate management of hospitalized patients requiring hemodialysis (HD) and/or ultrafiltration for end-stage renal disease (ESRD) or acute kidney injury (AKI), who frequently have mismatch between blood pressure (BP) and intravascular volume, or between extravascular and intravascular volume [1].

Critically ill patients with undifferentiated hypotension have been shown to benefit from inferior vena cava ultrasound (IVC US) assessment of relative intravascular volume to guide volume management [3,4]. IVC US may be a useful tool for predicting whether critically ill patients are likely to tolerate volume removal with hemodialysis (HD) and ultrafiltration (UF) [5]. In our previous study, critically ill patients with low inferior vena cava (IVC) collapsibility index (CI) values, consistent with relative intravascular volume overload, more frequently tolerated UF, while those with high IVC CI consistent with low relative intravascular volumes were much less likely to tolerate UF [5]. Assessing relative intravascular volume using inferior vena cava collapsibility determined by ultrasound may provide a useful tool in guiding volume management in critically ill patients receiving HD/UF.

CO has been well documented to increase >10% after volume administration in volume depleted patients [1,6]. CO has only been reported to increase >10% with HD and UF in two studies of congested patients with stable ESRD [7,8], in one study of critically ill patients with renal failure and clinical volume overload [9], and with UF alone in three studies of refractory/decompensated heart failure [10–12].

CO has been reported to decrease >10% after intermittent HD and/or UF in multiple studies of stable ESRD patients with or without a propensity for intradialytic hypotension (IDH) [8,13–27]. CO has been reported to be decreased in critically ill patients with acute hepatic and renal failure receiving vasopressors during intermittent hemodiafiltration without net UF[28], in patients with AKI associated with critical illnesses during HD with or without UF [29], and with sepsis and acute kidney injury (AKI) during continuous veno-venous hemofiltration (CVVH) without UF [30].

Intradialytic hypotension (IDH) may relate to cardiac stunning and decreased CO in stable ESRD patients [25,26], and may limit the volume of net UF removed in critically ill patients [5].

Changes in relative intravascular and extravascular volume may not be accurately determined from either changes in total body weight or net “fluid” balance [31]. The volume effects of different enteral and parenteral solutions administered and body fluid losses vary widely [32]. When estimating relative intravascular and extravascular volume contribution of different “fluid” inputs and losses from the body, it is important to consider their individual compositions, as well as the normal distribution of water and electrolytes and the size of the major fluid compartments in the body. Changes in CO with HD/UF in critically ill patients receiving intermittent or continuous hemodialysis (HD) therapy have not been previously related to initial relative intravascular volume assessed by IVC US and subsequent net “isonatremic volume equivalent” changes (equivalent volume effect of 0.9% saline).

We evaluated a group of critically ill patients who had relative intravascular volume assessed by IVC CI, and CO estimated by thermodilution, before and after intermittent HD and/or UF or at intervals during continuous HD with or without UF, to assess effects of net volume change on change in CO.

## Material and methods

### Population and study design

We retrospectively studied ICU patients between August 2012 and September 2018 who had CO assessed by thermodilution using a Swan-Ganz catheter before and after intermittent HD or during continuous venovenous hemodiafiltration (continuous HD), and had relative intravascular volume assessed by respiratory changes in inferior vena cava (IVC) collapsibility within 24 h prior to intermittent HD or prior to CO measurement during continuous HD (Tables 1 and 2).

**Table 1.** Patient characteristics.

	Intermittent HD only	Continuous HD only	Both intermittent and continuous HD
Patient data	*N* = 15	*N* = 4	*N* = 3
Age (years) [median, range]	[60, 41–87]	[58, 43–63]	[58, 49–82]
Gender [M, F]	[10, 5]	[3, 1]	[2, 1]
Primary disease states			
AKI	3	2	2
AKI/CKD	9	2	1
ESRD	3	0	0
Heart failure	11	3	2
Other cardiac disorders	13	3	2
Sepsis and/or shock	6	2	2
Acute respiratory failure	3	0	3
Other comorbidities^a^	15	1	3
Echocardiography^b^			
(Days between echo and first CO) [median, range]	[−6, −16 to +11]	[0, −1 to 0]	[−6, −11 to −3]
HFrEF < 40%	6	2	1
HFmrEF 40–50%	1	0	0
EF > 50%			
HFpEF^c^	2	0	0
Not-HFpEF^d^	4	0	2
Normal	2	2	0
Comorbidities			
Diabetes mellitus	10	1	3
Hypertension	10	1	3
Survival [Y, N]	[9, 6]	[2, 2]	[1, 2]

^a^Patients have multiple other primary medical disorders and comorbidities including pulmonary embolism, pulmonary HTN, volume overload, liver failure, balloon pump, bleeding, hematologic disorders and malignancy.

^b^Echocardiography study performed closest to the time of the first CO encounter.

^c^HFpEF criteria [33].

^d^Causes of non-HFpEF included severe mitral valve disease (*n* = 1), pulmonary embolism (*n* = 1), and pulmonary HTN (*n* = 4).

CO: cardiac output; HF: heart failure; pEF: preserved ejection fraction; mrEF: mid-range ejection fraction; rEF: reduced ejection fraction.

**Table 2.** Patient encounter data.

Encounter data	Intermittent HD (*N* = 37)	Continuous HD (*N* = 21)	*p* Value
Ventilated [Y, N]	[26, 11]	[19, 2]	0.0621 LLR
Vasopressors/inotropes [Y, N]	[28, 9]	[21, 0]	0.0027 LLR
Encounter SOFA score [median, range]	[13, 4–19]	[14, 11–19]	0.0083 MW
Severity of intradialytic hypotension			0.017 LLR
** 0** None	0	0	
** 1** Received volume resuscitation only	0	0	
** 2a** MAP < 65 mmHg without vasopressors	4 ↑	0 ↓	
** 2b** Pre-HD hypotension on a constant dose of vasopressors	14 ↓	16 ↑	
** 3** SBP decreased > 50 mmHg or MAP decreased > 20%	13	4 ↓	
** 4** Vasopressor initiated or dose increased, or dialysis stopped < 2 h	6 ↑	1^a^ ↓	
Net volume change between CO values (mL/kg) [median, range]	[−17.16, −43.14 to +14.9]	[−5.54, −37.49 to +23.55]	0.0069 MW
Interval between CO values (hr) [median, range]	[8, 3 to 32]	[4, 2 to 30]	0.0752 MW
Rate of net volume change (mL/kg/hr) [median, range]	[−2.34, −10.85 to +3.72]	[−1.25, −3.54 to +5.89]	0.2012 MW

↑Indicates that frequency was higher than expected by chance. ↓ indicates that frequency was lower than expected by chance.

^a^ Continuous HD stopped due to intractable hypotension.

CO: cardiac output; LLR: log likelihood ratio test; MW: Mann-Whitney test.

Approval for this study was obtained from the University of Southern California Institutional Review Board (HS-12-00383-CR002). Written informed consent was not required for the retrospective data collection and analysis since all ultrasound studies, Swan-Ganz catheter placements and measurements, and treatment plans were performed for clinical purposes.

Dialysis and/or ultrafiltration were performed using intermittent HD if tolerated or continuous venovenous hemodiafiltration, at the discretion of the attending nephrologist. Volume management decisions were made in a clinical context, not according to a research protocol, based on all available data, including Swan-Ganz and IVC US findings. We generally aimed to optimize intravascular volume in the absence of overriding considerations or clinical goals [1].

CO values were included if they were recorded before and after intermittent HD, or if they were available immediately before and after, or during continuous HD. Individual patients have different relative intravascular volumes from one encounter to another due to volume administration, volume losses and other factors, as well as varying changes in cardiac output. Each interval between CO measurements was considered as a separate encounter, since critically ill patients have continuous variations in relative intravascular volume and CO as well as multiple other parameters over time.

All patients had echocardiography studies available during the hospitalization. For patients who had multiple echocardiograms, the study closest in time to the first encounter was used.

### Ultrasound

Bedside IVC US was performed in real-time during daily bedside rounds without Valsalva or sniff maneuvers, since most patients were unable to perform these maneuvers, as previously described [5]. For continuous HD encounters, all were scanned while on HD except for one who had IVC US pre-HD (Supplemental Table 1). Longitudinal images of the IVC were obtained from an anterior substernal approach performed or directly supervised by the senior investigator (EMK) with a 3.5-MHz curvilinear probe on a portable ultrasound machine (LOGIQ e B12, GE Healthcare, Wauwatosa, WI 53226). All patients were imaged in the semi-recumbent position. Substernal IVC diameters were measured 1–2 cm from the junction with the right atrium or distal to the hepatic vein. A frame-by-frame image analysis was performed to identify maximum (IVCmax) and minimum (IVCmin) cava diameters with respiration/ventilation. Collapsibility Index (CI = (IVCmax – IVCmin)/IVCmax * 100%) was calculated for all encounters [1].

### Severity of IDH

Severity of IDH was categorized as previously reported [5], with the greatest severity recorded for each encounter. Increasing severity of IDH in our ICU patients were as follows: 0, No criteria for IDH; 1, received more than 500 mL 0.9% saline or received albumin IV to treat IDH (all patients receiving intermittent HD received 250 mL of 0.9% saline initially to prime the line and dialyzer and 250 mL of 0.9% saline at the completion of HD to return the blood and rinse the line and dialyzer) [5]; 2a, MAP < 65 mmHg with or without volume resuscitation during HD, no vasopressors/inotropes given, and HD not discontinued due to hypotension; 2b, pre-HD hypotension requiring vasopressors/inotropes, and a constant dose of vasopressors/inotropes given to maintain BP before and during HD/UF with or without volume resuscitation; 3, SBP decreased more than 50 mmHg or MAP decreased more than 20% with or without volume resuscitation and/or vasopressors/inotropes; 4, vasopressor/inotrope therapy initiated, dose increased, or dialysis stopped within 2 h due to intractable IDH with or without volume resuscitation.

### Calculation of “isonatremic volume equivalent” changes

Supplemental Figure 1 summarizes how we estimated the intravascular and extravascular volume contribution of different enteral and parenteral solutions administered, and body fluid losses. We used population-based mean sodium concentrations for body fluid losses [32]. Net UF volume changes during intermittent HD included only those directly related to the dialysis procedure and blood products, for the time during which dialysis was performed. Net volume changes between CO measurements included all “isonatremic” volume inputs and losses, including those during the dialysis procedure.

Isonatremic solutions, for example 0.9% saline, equilibrate across intravascular and extravascular compartments in proportion to the relative sizes of those compartments [32]. The free water contribution to intravascular and extravascular volume has been shown to be minimal [34,35]. To determine what proportion of fluids will distribute as isonatremic solution, the sodium concentration of fluids lost and gained can be divided by the sodium concentration of plasma water (normally 154 mEq/L), except for blood products [32]. For all analyses in this study, milliliters of fluid inputs and outputs were converted into “isonatremic volume equivalents” (equivalent volume effect of 0.9% saline) using the calculations in the Supplemental Figure 1.

Net intravascular plus extravascular volume effect of pRBC administration using an additive solution was estimated as (pRBC volume * 2.8) as 0.9% saline equivalents (Supplemental Figure 1) [36,37].

The intravascular volume effect of albumin peaks immediately after IV administration and decreases progressively with time [38]. The immediate intravascular volume effect of 25% albumin is approximately five times that of 5% albumin [39], and 5% albumin has a similar volume effect immediately after IV administration as 0.9% saline [40]. For net volume calculations which included 25% albumin, the intravascular volume effect was adjusted for the time from administration to the time of the CO measurement (Supplemental Table 1).

If urine sodium concentration was not measured, urine was assumed to be approximately 0.45% saline (77 mEq/L) with severe renal insufficiency or diuretics. If stool loss was indicated but no volume recorded, we assumed loss was approximately 100 mL. If there were stool losses but no lactulose, sorbitol or polyethylene glycol (PEG) had been administered, we assumed non-osmotic diarrhea with mean sodium concentration of 69 mEq/L [32]. Other body fluid losses were estimated as indicated in Supplemental Figure 1.

### Statistical analysis

Statistics were performed using Epistat version 5.3 (Epistat Services, Richardson, TX, USA) and graphics were created using SigmaPlot version 13 (Systat Software Inc, San Jose, CA, USA).

Category variables were analyzed using log likelihood ratio tests (LLR). Values for numeric variables were compared using non-parametric Mann-Whitney U test (MW) when data was distributed between two categories, and using non-parametric Kruskal-Wallis ANOVA (KW) test with multiple categories. When the Kruskal-Wallis test indicated significance, Newman-Keuls (NK) multiple comparisons tests at the *p* = 0.05 significance level were used to determine which groups differed.

**Figure 1.**
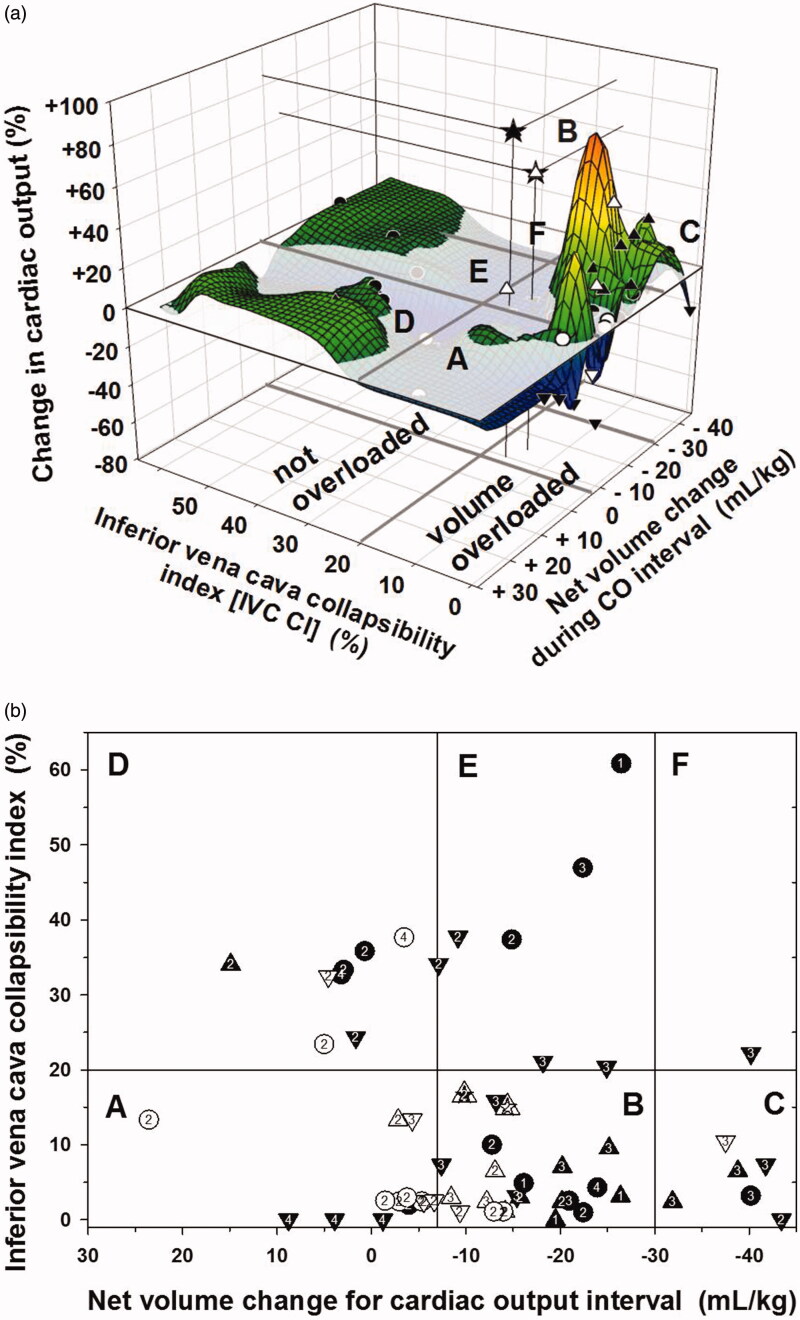
(a) Relationship of change in CO to “net volume” change within CO interval and to IVC CI depicted as a 3-D scattergram with smoothing. Solid symbols indicate intermittent HD encounters and open symbols indicate continuous HD encounters. Upward triangles indicate CO increased > 10%, downward triangles indicate CO decreased > 10%, circles indicate CO changed −10% to +10%. Stars indicate CO “outliers” on the mesh plot. IVC CI < 20% was indicated as relative intravascular volume overload and IVC CI ≥ 20% as not volume overloaded. The flat plane indicates zero per cent change in CO. Sectors labeled A, B, C, D, E, and F correspond to Figure 1(b). (b) Relationship of IVC CI to “net volume” change between CO measurements, CO change, and intradialytic hypotension. Symbols are as in Figure 1(a). Encounter data are grouped based on IVC CI <20% versus ≥20%. The “net volume” change cutoffs were arbitrarily at −7 mL/kg and −30 mL/kg. The numbers inside of the symbols indicate the severity of intradialytic hypotension (IDH) (number 1 = IDH 2a, 2 = IDH 2b, 3 = IDH 3, and 4 = IDH 4). For IVC CI < 20%, sector A represents “net volume” change of < −7 mL/kg (*n* = 12), sector B represents “net volume” change −7 to −30 mL/kg (*n* = 24), and sector C represents “net volume” change of more than −30 mL/kg (*n* = 6). For IVC CI ≥ 20%, D represents “net volume” change of < −7 mL/kg (*n* = 8), E represents “net volume” change −7 to −30 mL/kg (*n* = 7), *F* represents “net volume” change of more than −30 mL/kg (*n* = 1).

The change in CO with net “isonatremic volume equivalent” change (abbreviated as “net volume” change) may depend on initial relative intravascular volume. The relationship of change in CO to “net volume” change between cardiac measurements and to IVC CI was plotted using a 3-D scattergram with smoothing (Figure 1(a)). CO change >10% was considered significant [6].

We plotted IVC CI versus weight-adjusted “net volume” change for cardiac output intervals, and divided encounters into categories by IVC CI and “net volume” change (Figure 1(a,b)). We used IVC CI cutoff < 20% and ≥20% as previously published [5], and arbitrary “net volume” change cutoffs of more than 30 mL/kg, and <7 mL/kg derived from natural breaks in the data. Changes in CO were categorized as >+10%, −10% to +10%, and < −10%. The severity of intradialytic hypotension is indicated for each encounter (Figure 1(b) and Supplemental Table 1). All summations for contingency table analyses can be derived from Supplemental Table 1.

Sequential Organ Failure Assessment (SOFA) scores were calculated for each encounter [41] (Supplemental Table 1). Relationships between encounter SOFA scores with IVC CI values were assessed by linear regression, and between SOFA scores and changes in CO (as increased >10% or not increased) were determined by Mann-Whitney U test.

## Results

As shown in Table 1, CO data were available for 15 patients who received intermittent HD and/or UF, 4 who received continuous HD and/or UF, and 3 who had both intermittent and continuous HD and/or UF encounters. There were 37 encounters with intermittent and 21 with continuous HD and/or UF (Table 2).

As shown in Table 2, significantly more encounters with vasopressor/inotrope support occurred with continuous HD (100%) compared to intermittent HD (75.7%) (*p* < 0.003), and SOFA scores were higher in the continuous than intermittent HD encounters (*p* < 0.009). The number of encounters with IDH 2a and IDH4 were higher than expected due to chance in intermittent HD and the number of encounters with IDH 2 b was higher than expected in the continuous HD encounters (*p* = 0.017 LLR). The weight-adjusted “net volume” change between CO measurements was larger in the intermittent than the continuous HD encounters (*p* < 0.007), while the rate of “net volume” change was not significantly different for intermittent than continuous HD encounters (*p* = 0.2) which may be due to the tendency for longer intervals between CO measurements with intermittent HD (*p* = 0.075).

Encounters were grouped based on IVC CI < 20% versus ≥ 20%, and changes in CO of greater than 10% increase −10% to +10%, and greater than 10% decrease (Table 3). There were 42 encounters with IVC CI < 20% (24 intermittent and 18 continuous HD) and 16 encounters with IVC CI ≥ 20% (13 intermittent and 3 continuous HD). Details of each encounter are presented in Supplemental Table 1.

**Table 3.** Summary of patient encounter data related to inferior vena cava collapsibility and cardiac output categories.

Change in CO category	*N*	IVC CI (%)	Change in CO (%)	Net volume change between CO values (mL/kg)	Rate of net volume change between CO values (mL/kg/hr)
		(Median, range)	(Median, range)	(Median, range)	(Median, range)
Relative intravascular volume overloaded (IVC CI <20%)					
Increased > 10%	15	3.2	+25.2	−15.7	−2.52
		(0 to 16.7)	(+12.6 to +85.1)	(−38.8 to −2.8)	(−5.54 to −0.49)
−10% to +10%	14	2.5	+0.26	−13.0	−2.73
		(1.0 to 13.3)	(−7.7 to +9.4)	(−40.2 to +23.6)	(−5.02 to +5.89)
Decreased > −10%	13	2.5	−19.4	−8.4	−1.39
		(0 to 15.8)	(−56.5 to −13.2)	(−43.4 to +8.8)	(−10.85 to +1.02)
Not relative intravascular volume overloaded (IVC CI ≥ 20%)					
Increased > 10%	1	34.1	+12.6	+14.9	+3.72
−10% to +10%	8	36.6	+2.35	−3.5	−0.10
		(23.4 to 60.9)	(−7.7 to +8.1)	(−26.5 to +3.2)	(−3.43 to +1.07)
Decreased > −10%	7	24.3	−16.2	−9.2	−1.02
		(20.4 to 37.7)	(−44.4 to −11.4)	(−40.2 to +4.6)	(−6.23 to +0.62)

CO: cardiac output; IVC CI: inferior vena cava collapsibility index; *N*: number of encounters.

Figure 1(a) shows the 3-D mesh plot of the relationship of change in CO to relative intravascular volume assessed by IVC CI before each CO interval and to weight-adjusted “net volume” change during intermittent or continuous HD. A similar profile was seen using mean rate of “net volume” change during CO intervals (Not shown).

Figure 1(b) shows the 2D scatter plot of IVC CI < 20% and ≥ 20% versus “net volume” change categories (more than −30, −30 to −7, and less than −7 mL/kg) and also shows changes in CO and severity of IDH for each encounter (data from Supplemental Table 1).

CO increased >10% in 15 of 42 encounters with IVC CI < 20% and in 1 of 16 encounters with IVC CI ≥ 20% (*p* = 0.0136 LLR) (Table 3). CO decreased >10% in 13 of 42 encounters with IVC CI < 20% and in 7 of 16 encounters with IVC CI ≥ 20% (*p* = 0.35 LLR), and change in CO was between −10% and +10% in 22 of 42 encounters.

Of the 16 encounters with increased CO >10%, 15 had IVC CI < 20% (Sectors A, B and C) and one had IVC CI of 34% (Sector D) (Figure 1(a,b)). Those with IVC CI <20% and increased CO achieved “net volume” changes ranging from −39 mL/kg to −9 mL/kg. The encounter with IVC CI of 34% and increased CO >10% had a “net volume” change of +15 mL/kg (Sector D) (Supplemental Table 1).

The distribution of IDH severity in encounters with IVC CI <20% was not random among “net volume” change categories (*p* = 0.0095 LLR) (Figure 1(b)). In encounters where only minimal “net volume” was removed or volume was added (Figure 1(b) sector A), there was higher than expected number of encounters with IDH category 4 and a lower than expected number of encounters with IDH category 3. In encounters where the largest “net volume” was removed (Figure 1(b) sector C), there was a higher than expected number of encounters with IDH category 3.

**Figure 2.**
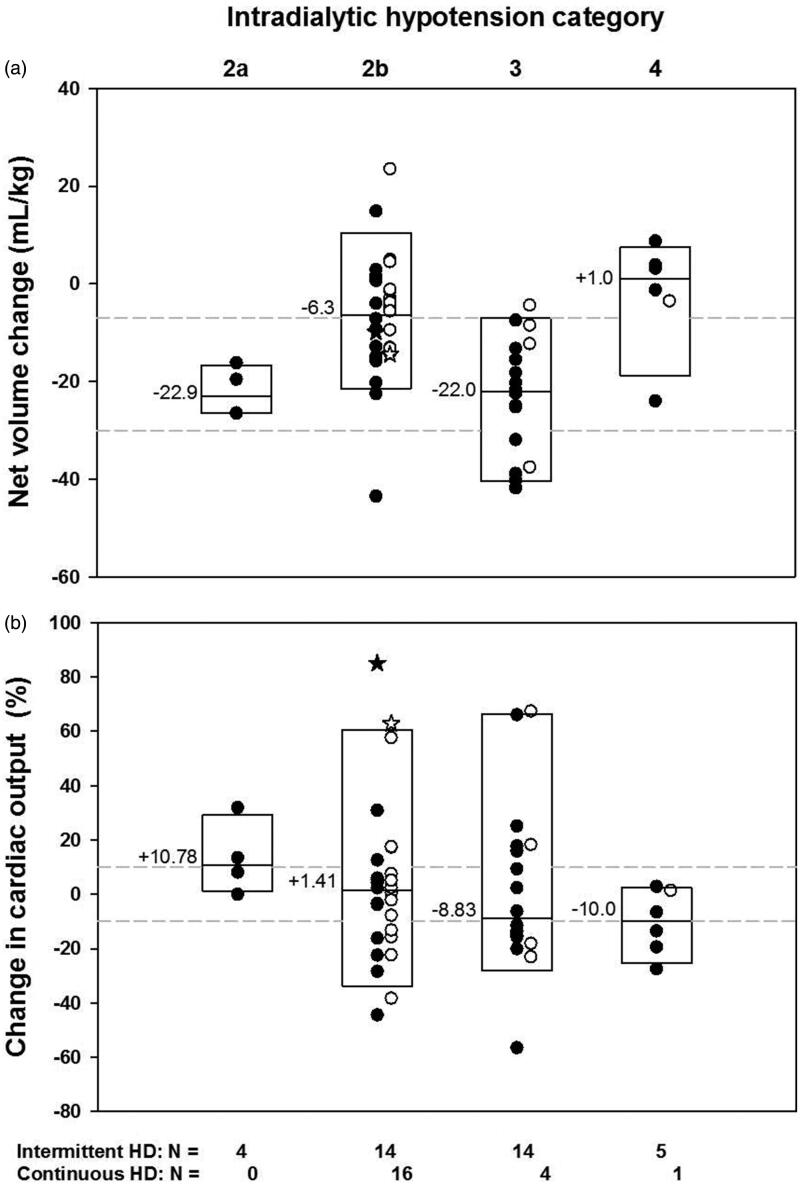
Relationship of net volume change between CO measurements to severity of IDH (Figure 2a) and **t**he relationship between changes in CO values with severity of IDH (Figure 2(b)). Solid symbols indicate intermittent HD encounters and open symbols indicate continuous HD encounters. Stars indicate CO “outliers” on the mesh plot. Boxes indicate the 95th, 50th and 5th percentiles. Median values are indicated. The number of encounters is indicated as N. (a) The weight-adjusted median net volume changes were less with IDH 4 than with IDH 2b, which were less than with IDH 2a or IDH 3 (KW *p* < 0.001, NK at *p* of 0.05). The dashed lines represent the “net volume” change cutoffs of −7 mL/kg and −30 mL/kg. (b) An increased CO > 10% was more frequent with IDH 2a and less frequent with IDH 4 than expected due to chance. A decreased CO more than 10% was less frequent with IDH 2a and IDH 2b, and more frequent with IDH 3 and IDH 4 than expected due to chance (*p* = 0.047 LLR) (Supplemental Table 2). Median values were not significantly different.

The median “net volume” change between CO measurements for the combined intermittent and continuous HD encounters were less with IDH 2b and IDH 4 than with IDH 2a and IDH 3 which did not differ from each other; the “net volume” change was less with IDH4 than with IDH 2 b (*p* < 0.001 by KW) (Figure 2(a)). The same pattern was present for the median rates of net volume change among IDH categories (*p* < 0.001) (Data not shown). The net UF volumes and UF rates for the intermittent HD encounters were significantly less with IDH 4 than with IDH 2a, IDH 2b or IDH 3 which were not different from each other (*p* = 0.004 for UF volumes, and *p* = 0.006 for UF rates, respectively) (Data not shown).

The distribution of encounters with varying severity of IDH and change in CO as increased, unchanged or decreased, was not random (*p* = 0.047 by LLR) (Figure 2(b) and Supplemental Table 2). For those encounters with more than 10% increase in CO, IDH 2a was more frequently observed than expected and IDH 4 was less frequently observed than expected. For those encounters whose CO was decreased more than 10%, IDH 3 and IDH 4 were observed more frequently than expected and IDH 2a and 2b were observed less frequently than expected (Supplemental Table 2). Median changes in CO were not significantly different among IDH categories. The changes in SVR or 1/SVR were not significantly related to IDH categories (Data not shown).

Changes in CO were linearly correlated directly with changes in stroke volume (*R*^2^ = 0.82, *p* < 0.001) and to changes in 1/SVR (*R*^2^ = 0.49, *p* < 0.001). There was no relationship between a diagnosis of cardiac disease and the number of encounters with increased, decreased or no change in CO (*p* = 0.16 LLR), or for those with increased CO compared to those with no increase in CO (*p* = 0.15 LLR). There was no significant relationship between the frequency of encounters for CO increased or not increased when comparing numbers of encounters with moderate to severe mitral regurgitation to those without moderate to severe mitral regurgitation (*p* = 0.30 LLR) (Supplemental Table 1). The distribution of the frequency of increased CO or not increased with or without administration of vasopressors/inotropes including dopamine, dobutamine and epinephrine, which may increase CO [42], was not different from random in either the intermittent HD (*p* = 0.07) or continuous HD encounters (*p* = 1.0), or for the combined group (*p* = 0.14) (Supplemental Table 1). Encounter SOFA scores were not significantly related to whether change in CO was increased or not increased.

Among encounters with IVC CI <20%, the frequency of increased CO >10% compared to no change or decreased CO was not significantly different for baseline diagnoses including cardiac diseases (*p* = 0.156) or sepsis (*p* = 0.094) (Data from Supplemental Table 1). There was no difference in frequency of severity of IDH (*p* = 0.166), or mechanical ventilation (*p* = 0.54), or after excluding the multiple encounters for patient #1 (*p* = 0.292 and *p* = 1.0, respectively). The mean net UF volume changes (mL/kg) or median SOFA scores did not differ between those in whom CO increased >10% or not for all encounters (*p* = 0.179 and *p* = 0.341, respectively), or for encounters excluding patient #1 (*p* = 0.463 and *p* = 0.932, respectively). The frequency of increased CO was not significantly different with administration of vasopressors/inotropes which affect CO (*p* = 0.105) but was significantly more frequent when encounters for patient #1 were excluded (*p* = 0.018 LLR).

There were no significant relationships between IVC CI and right atrial pressure (RAP) (*p* = 0.25), PAOP (*p* = 0.058) or SVR (*p* = 0.89), or between change in weight-adjusted “net volume” and change in RAP (*p* = 0.38) or change in PAOP (*p* = 0.058). There was a significant relationship between absolute change in RAP and absolute change in PAOP in all encounters (*R*^2^=0.30, *p* < 0.001) as well as for encounters with increased CO (*R*^2^=0.32, *p* = 0.02). There was no association between the frequency of IVC CI <20% or ≥20% and the presence or absence of moderate to severe tricuspid regurgitation by echocardiography (*p* = 0.53 LLR).

## Discussion

CO increased >10% in 36% of our critically ill patient encounters with relative intravascular volume overload asessed by IVC CI <20% followed by net volume removal during intermittent or continuous HD, while CO decreased ≥10% in 34%, and was between −10% and +10% in the remaining 30%. CO increased in only one encounter with IVC CI ≥20% after volume administration (Table 3).

In prior studies, CO was shown to increase after HD with or without UF or after UF alone in 11 reports and was decreased or unchanged in 36 reports (Table 4). For studies in which the CO increased, most patients were clinically assessed to be volume overloaded, while for the majority of studies in which CO decreased or was unchanged, volume status varied or was not reported.

**Table 4.** Review of the literature of changes in CO with hemodialysis with or without ultrafiltration.

Author/year	Study design	Number of pts/HDs	Reason for HD/UF	Pre HD/UF volume	Procedure	Post HD/UF volume	Mean volume of UF (L)	% Change in mean CO (% of patients)
Cardiac output increased > 10%								
Del Greco 1964 [7]	Observational	9	Hospitalized CKD	Congested	HD/UF	TBW ↓2kg BV ↓12%, PV ↓17%	Not available	↑46%
Del Greco 1969 [8]	Observational	31	Hospitalized CKD	Congested	HD/UF	TBW ↓2.2 to 3.0 kg (↓3–4%)	Not available	↑55% (↑ in 19%)
Wehle 1979 [13]	Prospective observational	7	Stable ESRD	(4 of 7 IDH, 3 CHF)	HD only	TBW no change	No UF	↑18–22%
Lauer 1988 [9]	Observational	9/10	AKI or CKD	Volume overloaded	CAVH (UF)	Improved, TBW ↓7 kg (↓8%)	−7 net volume loss	↑26% (↑ in 90%)
Fox 1993 [43]	Randomized Cross over	9	Stable ESRD		HD/UF HF/UF	TBW ↓2.7kg (↓3.6%) TBW ↓2.6kg (↓3.7%)	Not available	↑28% ↓3%
Heering 1997 [30]	Prospective controlled	15	Non-septic CVD and AKI	Volume overloaded	CVVHF only		No UF	↑12% at 24 hr
Honore 2000 [44]	Prospective STHVH 4 hr	11 responders 9 non responder	Refractory septic shock AKI		Responders Non-responders		0 0	↑147% ↓3%
Marenzi 2001 [12]	Observational	24	Refractory CHF	Congested	SCUF	PAOP ↓20%	−4	↑20%
Giglioli 2010 [10]	Observational	15	ADHF	Hypervolemia	SCUF	TBW ↓6kg (↓8%)	−3.1 net fluid balance	↑14%
Giglioli 2011 [11]	RCT	15	ADHF	Congested	SCUF	Improved, TBW↓7%	Not available	↑23%
Jeong 2018 [45]	RCT crossover of exercise	12	Stable ESRD No exercise	Not reported	HD/UF No exercise	IDH in 67% TBW ↓2.4kg (↓4%)	−3.1	↑11%
Cardiac output decreased > −10%								
Goss 1967 [27]	Observational	9	Stable ESRD	No congestion	HD/UF	RAP ↓9 to 4 mmHg	Not available	↓29%
Del Greco 1969 [8]	Observational	37	Hospitalized CKD	No congestion	HD/UF	PV↓12%	Not available	↓11%
Wehle 1979 [13]	Prospective Observational	7	Stable ESRD	4 IDH, 4 HF	UF only	TBW ↓1.7–1.9 kg (↓3 % )	Not available	↓35–41%
Rouby 1980 [14]	Observational	10	Stable ESRD	4 of 8 had ↑PV	UF only HD/UF	PAOP↓14–7 mmHg PAOP ↓10–6 mmHg IDH in 80%	−2.0 to −2.2	↓42% ↓30%
Chaignon 1982 [46]	Observational	5/8	Stable ESRD	Not reported	HD/UF HF/UF	TBW↓3.6 (↓6%) TBV ↓6.7 TBW↓3.9 (↓6%) TBV ↓ 6.7	Not available	↓16.1 ↓6.1
Davenport 1993 [28]	RCT	12 IHF 20 CRRT	Acute liver and renal failure	Goal: isovolemia	IHF CRRT	RAP↓12–7 mmHg RAP 12–12 mmHg	No UF No UF	↓15% ↓3%
Pepi 1993 [47]	RCT	12 SCUF 12 no SCUF	Heart failure	Hypervolemia	SCUF	RAP ↓50%	−1.9 0	↓17%
Wakabayashi 1994 [29]	RCT Prospective	8	Critically ill AKI	Not reported	HD/UF	PAOP unchanged PAOP unchanged	−0 to −3	↓ 15% HCO_3_^–^↑12% acetate
Heering 1997 [30]	Prospective controlled	18	AKI with sepsis	Volume overloaded	CVVHF		No UF	↑4% at 12 hr ↓13% at 24 hr
Bos 2000 [15]	Prospective observational	9	ESRD	Not reported	UF HD/UF	TBV ↓4% ↓7%	−0.5 −1.5	↓13% ↓13%
Hoeben 2002 [20]	Prospective controlled	14/48	ESRD, Heart disease	Intractable IDH	HD/UF only Cool dialysate Midodrine	CBV ↓13% and ↓28% ↑76% ↑6%	−3.3 to −3.5	↓27 and ↓14% ↓7% ↓23%
Gadegbeku 2003 [16]	Prospective observational	27	Stable ESRD	Not reported	HD/UF		−3.0	↓19%
Boon 2004 [17]	Prospective observational	19	Stable ESRD	Clinical “Euvolemia”	HD/UF	No IDH, BV ↓10.6–11.2%	−2.1 to −2.3	↓23–39%
Karamperis 2005 [18]	RCT	12	Stable ESRD	Not reported	HD/UF HDF/UF	RBV ↓10% RBV ↓11%	−2.9 to −3.1	↓20% ↓19%
Yoshii 2005 [19]	Prospective observational	19	Stable ESRD	Not reported	HD/UF	No IDH BV↓16%, Hct↑20%	Not available	↓35%
Karamperis 2005 [48]	RCT	12	Stable ESRD	Not reported	HF/UF	RBV ↓88–89%	−2.9 to −2.9L	↓21–22%
Selby 2006 [21]	RCT crossover	8/64	ESRD, LVH	Prone to IDH	HD/UF Biofeedback HD	RWMA 88–100% IDH in 38–78%	−1.5 to −1.9	↓18–26% with stunning
Chou 2006 [49]	RCT	30 HTN 30 controls	Stable ESRD	Prone to HTN	HD/UF HTN HD/UF controls	Hct ↑11% Hct ↑18%	−2.1 −2.3	↓16% ↓11%
Dasselaar 2009 [22]	Observational	7	Stable ESRD	No IDH	HD/UF	↓ myocardial blood flow	−1.3 to −3.8 Mean −2.8	↓21% (↓ in 5 of 7 pts)
Yang 2010 [23]	Prospective observational	29 34	ESRD	With IDH No IDH	HD/UF		−2.2 to −2.9 −1.6 to −2.2	↓18% ↓6%
Cornelis 2014 [24]	RCT	13	Stable ESRD	Volume overload	HD4/HDF4 HD8/HDF8	RBV↓8.1–9.1% ↓3.3–4.4%	−1.8 to −2.0	↓22–24% ↓6–8%
Maarek 2016 [25]	Prospective observational	24/64	ESRD prone to IDH	BV 8% lower with IDH pre-HD	HD/UF With IDH No IDH	IDH in 31% CBVI ↓13–16% Hct ↑3%	−3.3 to −4.4 −3.0 to −4.0	↓30% ↓19%
Schmidt 2016 [50]a	Prospective observational	11/17	Critically ill	Not reported	10 IHD 7 CVVH	ITBV ↓17–21%		↓17–25%
Buchanan 2017 [26]	RCT, crossover	12	ESRD	Not reported	HD/UF HDF/UF	IDH in 1 of 24 Stunning in 85%	−1.1 −1.3	↓27% ↓34%
Czyzewski 2017 [51]	Observational multicenter	27 27	ESRD CVD (80%)	Not reported	HD/UF 4 hr HD/UF 5 hr		−1.8 −2.8	13% ↓ 7%
Cardiac output changed −10 to +10%								
Ikaheimo 1981 [52]	Observational	41/69	Stable ESRD	Not reported	HD/UF	TBW ↓1.2 kg (↓2%)	Not available	↓6%
Teo 1987 [53]	RCT cross over study	10	Stable ESRD	Not reported	HD/UF acetate HDF/UF acetate	TBW ↓2.8 kg (↓4%) ↓3.0 kg (↓4%)		↓11% ↓9%
Rimondini 1987 [54]	Prospective observational	11	CHF	Hypervolemic	HF/UF	PAOP ↓23 to 12mmHg RAP ↓11 to 3mmHg	−2 to −3 Mean −2.45	No change
Susini 1990 [55]	Prospective observational	20	CHF	Hypervolemic	SCUF	PAOP ↓29 to 7mmHg RAP ↓14 to 5mmHg	−3.0	No change
Barnas 1999 [56]	Observational	26 22	Stable ESRD	Not reported	HD/UF no IDH HD/UF with IDH		−1.9 −2.6	↓8% ↓12%
Klouche 2002 [57]	Prospective observational	11	Sepsis, AKI	Not reported	CVVHDF	Improved symptoms TBW unchanged	No UF	No change
Prakash 2002 [58]	Prospective observational	20	ESRD, stable	Hypervolemic	HD/UF	TBV ↓7.1% TBW ↓4.6%	Not available	No change
Ratanarat 2005 [59]	Prospective observational	15	Sepsis	Not reported	PHVHF/UF CVVHF		Not available	No change
Schytz 2015 [60]	RCT crossover	22	Stable ESRD	No IDH	HD/UF then HD only		−1.5	Unchanged
Sarafidis 2017 [61]	RCT crossover	41	Stable ESRD	Not reported	HD/UF after 3d HD/UF after 2d	TBW ↓2.9kg (↓4%) RAP↓19% TBW ↓2.7kg (↓4%) RAP ↓33%	Not available	↓6% ↓4%
Haag 2018 [62]	Prospective multicenter	215	Stable ESRD	Not reported	HD/UF	IDH in 32% Stunning in 3.7% CBV ↓11%	−2.1 to −6.5	↓10% (↓20% in 28%)

Inclusion criteria: CO measurements available before and after HD, UF and/or HD/UF. Exclusion criteria: No data presented for CO.

^a^ May be erroneous decrease in CO measurement if injectable indicator is injected into dialysis catheter.

ADHF: acute decompensated heart failure; AKI: acute kidney injury; BV: blood volume; CAVH: continuous arteriovenous hemofiltration; CBV: central blood volume; CHF: congestive heart failure; CKD: chronic kidney disease; CO: cardiac output; CRRT: continuous renal replacement therapy; CVD: cardiovascular disease; CVVHF: continuous veno-venous hemofiltration; ECF: extracellular fluid; ESRD: end stage renal disease; HD/UF: hemodialysis/ultrafiltration; HD4 : 4 h hemodialysis; HD8 : 8 h hemodialysis; HDF4 : 4 h hemodiafiltration; HDF8 : 8 h hemodiafiltration; HF: hemofiltration; hr: hour; IDH: intradialytic hypotension; IHF: intermittent hemofiltration; ITBV: intrathoracic blood volume; N: number of patients; PAOP: pulmonary artery occlusion pressure; PHVHF: pulse high volume hemofiltration; pts: patients; PV: plasma volume; RBV: relative blood volume; RCT: randomized control trial; SCUF: slow continuous ultrafiltration; STHVH: short-term high-volume hemofiltration; TBV: total blood volume; TBW: total body weight; UF: ultrafiltration.

IVC CI may reflect relative intravascular volume and provide a clinical assessment of the patient’s ability to respond to volume removal. All encounters in which CO increased with “net volume” removal had IVC CI <20% consistent with relative intravascular volume overload; one encounter with IVC CI of 34%, not consistent with relative volume overload, had an increase in CO after “net volume” administration. All other encounters with IVC CI ≥20% had a decrease or no change in CO after HD/UF. This suggests that if the IVC CI is <20%, indicating relative intravascular volume is increased, CO may improve with volume restriction and “appropriate” UF.

An increase in CO in 36% of our patient encounters who had increased relative intravascular volume prior to HD/UF may relate to “appropriate” volume removal, also shown in patients with heart failure following ultrafiltration (Table 4). CO has also been well documented to increase in patients without volume overload with “appropriate” net volume administration [6], as was noted in one of our patient encounters.

In studies reporting increased CO after HD and/or UF, the patients tended to be “congested” or “overloaded”, but not all patients had increased CO with volume removal. In one report, CO increased in only 19% of hospitalized patients after dialysis/UF, who were assessed to have volume overload and severe chronic renal failure; CO did not increase in any of the patients without “congestion” after HD/UF [8]. CO increased in 9 of 10 patients with volume overload and acute or chronic renal failure following continuous HD/UF [9], and mean CO increased >10% in patients with decompensated heart failure after UF [10–12]. In patients with refractory congestive heart failure, cardiac output has been postulated to increase after volume removal with UF due to reduction of extracardiac constraint; the latter has been attributed to increased lung edema, pleural effusions and ascites, which in turn may decrease right and left ventricular filling pressures [12].

Inotropes/vasopressors such as dopamine, dobutamine and/or epinephrine may increase CO [42], and may have played a role in a subset of our patient encounters with relative intravascular overload.

CO was decreased or unchanged in 64% of our patient encounters. In the majority of reported studies, CO after HD/UF was unchanged or decreased and patients were frequently clinically “not congested” or “prone to intradialytic hypotension” (Table 4). Relative intravascular volume status was not evaluated pre-HD/UF in most reported studies although some studies reported mean right atrial pressure (RAP) or pulmonary artery occlusion pressure (PAOP) values prior to HD/UF (Table 4).

In our critically ill patients, all HD encounters met criteria for IDH. In stable ESRD patients, larger UF volumes and more rapid UF rates have been associated with more frequent intradialytic hypotension [63], decreased CO during HD with or without intradialytic hypotension [21,22,26,62], as well as reduced myocardial blood flow and myocardial stunning [26,63–65]. In our study, encounters with the most severe IDH (IDH 4), had minimal changes in the net volume and rate of net volume removal, and the largest rates of net volume change were observed in encounters with less severe IDH. These findings suggest that the most severe IDH may have limited the amount of net volume removal rather than large net volume removal causing more severe IDH.

In our patient encounters, a cardiac output increase >10% was more frequent with the least severe intradialytic hypotension and was less frequent with more severe intradialytic hypotension. IDH may have induced myocardial stunning and contributed to decreased CO in some encounters. The frequency of CO changes relative to IDH severity suggests that more severe intradialytic hypotension may have played a role in decreasing CO in our patient encounters, which may in turn have been related to myocardial stunning as previously reported [63]. These relationships of changes in “net volumes” and changes in CO with categorization of severity of IDH suggests that our IDH categorization is clinically relevant.

Multiple definitions of intradialytic hypotension have been proposed, without consensus or definition of severity [66,67]. We previously categorized severity of IDH based on the degree of hypotension, requirements for volume resuscitation, use of vasopressors, and ability to continue the HD procedure [5]. We defined IDH 4 as initiation or increase of vasopressor/inotrope therapy or discontinuation of dialysis within 2 h due to intractable IDH with or without volume resuscitation. In our previous study [5], encounters with IDH 4 had vasopressors started or increased in 32 out of 33 encounters, while in the current study, those with IDH 4 had HD terminated early due to intractable hypotension in 3 out of 4 encounters. This suggests our IDH 4 criteria may have to be further defined as IDH 4a, indicating intractable hypotension requiring initiation of vasopressors or an increase in the vasopressor dose, and IDH 4b indicating the additional necessity of terminating HD/UF early due to intractable hypotension.

### Limitations

This was a retrospective observational study that was not controlled, randomized or blinded. Multiple factors could affect the IVC CI and the changes in CO. The number of patients and encounters was small due to the infrequent concurrent clinical use of Swan-Ganz catheters and IVC ultrasound. IVC ultrasound measurements were not performed immediately prior to CO measurements or immediately prior to and after HD. CO measurements were not available immediately prior to and after intermittent HD. Some patients had multiple encounters which may have been interdependent and may have introduced bias.

## Conclusion

In this study, despite the severity of illness and occurrence of IDH, CO increased after intermittent or continuous HD/UF with net volume removal in a third of encounters assessed to have relative intravascular volume overload by IVC ultrasound. This suggests that “appropriate” volume removal may be associated with increased CO in a subset of volume overloaded critically ill patients.

The most severe IDH was associated with the lowest UF volumes and rates, and with the smallest changes in “net volume” and lowest rates of “net volume” removal between CO measurements, suggesting that severity of IDH may have limited volume removal, rather than larger/more rapid volume removal being associated with more severe IDH.

## Supplementary Material

Supplemental Material

Supplemental Material
