# Supplementary material for: Changes in cardiac output with hemodialysis relate to net volume balance and to inferior vena cava ultrasound collapsibility in critically ill patients
Source: Ren Fail. 2020 Feb 12;42(1):179–92. doi: 10.1080/0886022X.2020.1726384 (PMC7034082; doi:10.1080/0886022X.2020.1726384)
Supplement: Supplemental Material [file IRNF_A_1726384_SM5543.docx]

| Supplemental Table 1: Individual encounter data for intermittent and continuous hemodialysis | | | | | | | | | | | | | | |
| --- | --- | --- | --- | --- | --- | --- | --- | --- | --- | --- | --- | --- | --- | --- |
| Patient ID number:days after initial encounter | IHD or CRRT encounter | IVC CI (%) | Group (As per Figure 1b) | Net IHD UF volume including pRBCs  (mL/kg) | Net rate of UF with IHD (mL/kg/hr) | Net volume change between CO values (mL/kg) | Rate of net volume change between CO values (mL/kg/hr) | Change in CO (%) | Vasopressors/inotrope dose (mcg/kg/min)  Vasopressin (milliunits/kg/min) | Inotrope effect on CO [^41^](#_ENREF_41) | Category of IDH severity ^b^ | Mechanical ventilation | SOFA score | Disease sates ^c^ |
| RELATIVE INTRAVASCULAR VOLUME OVERLOAD  **IVC CI < 20% with increase in CO > 10% (n=15)** | | | | | | | | | | | | | | |
| Pt 1: 11.2^d^ | IHD^a^ | 6.5 | C | -46.2 | -13.19 | -38.8 | -5.54 | +25.2 | Epi 0.05 | ↑↑↑ | 3 | N | 13 | 1,4 |
| Pt 2: 6.8 | IHD | 2.4 | C | -41.2 | -9.48 | -31.9 | -3.54 | +17.8 | Dopa 6 | ↑↑ | 3 | N | 12 | 2,4,6,8 |
| Pt 3: 0.0 | IHD^a^ | 3.2 | B | -28.8 | -7.21 | -26.4 | -3.30 | +30.1 |  |  | 2a | N | 9 | 2,4,5,8d |
| Pt 2: 0.0 | IHD | 9.6 | B | -29.8 | -7.46 | -25.2 | -3.15 | +66.1 | Dopa 15 | ↑ | 3 | Y | 16 | 2,4,6,8c |
| Pt 2 3.0 | IHD | 7.1 | B | -25.3 | -6.20 | -20.2 | -2.53 | +16.0 | Dopa 12 | ↑ | 3 | Y | 14 | 2,4,6,8c |
| Pt 2: 6.0 | IHD | 2.4 | B | -25.3 | -8.43 | -20.2 | -2.52 | +30.9 | Dopa 4 | ↑↑ | 2b | N | 12 | 2,4,6,8 |
| Pt 1: 6.8 | IHD | 0.0 | B | -29.0 | -8.28 | -19.5 | -2.34 | +13.5 |  |  | 2a | Y | 7 | 1,4,8a,c |
| Pt 4: 0.0 | IHD | 3.0 | B | -39.5 | -9.88 | -15.7 | -0.49 | +12.6 | Norepi 0.11 | 0 | 2b | Y | 12 | 3,4,5,6,8b,c,e |
| Pt 5: 0.0 ^e^ | CRRT | 15.0 | B |  |  | -14.4 | -1.25 | +62.9 | Norepi 0.06  Vaso 0.99 | 0  0 | 2b | Y | 14 | 2,4,5,8b,c,d |
| Pt 1: 1.8 | CRRT | 1.1 | B |  |  | -14.2 | -3.54 | +57.7 | Norepi 0.12 | 0 | 2b | Y | 15 | 1,4,8a,c |
| Pt 1: 10.6 | CRRT | 6.5 | B |  |  | -13.1 | -3.27 | +17.6 | Epi 0.08 | ↑↑↑ | 2b | Y | 14 | 1,4 |
| Pt 1: 12.9^d^ | CRRT | 2.4 | B |  |  | -12.2 | -2.04 | +18.4 | Epi 0.01  Vaso 0.44 | ↑↑↑  0 | 3 | Y | 14 | 1, 4 |
| Pt 6: 3.0 ^e^ | IHD | 16.7 | B | -17.2 | -4.30 | -9.8 | -1.23 | +85.1 | Norepi 0.08  Dopa 10  Dobut 3 | 0  ↑↑  ↑↑ | 2b | Y | 11 | 2,4,7, 8c,d |
| Pt 7: 0.25 | CRRT | 3.0 | B |  |  | -8.4 | -1.01 | +67.5 | Epi 0.04  Dobut 5 | ↑↑↑  ↑↑ | 3 | Y | 14 | 2,4,8c,d |
| Pt 1: 10.1 | CRRT | 13.3 | A |  |  | -2.8 | -0.71 | +17.4 | Epi 0.09 | ↑↑↑ | 2b | Y | 16 | 1,4 |
| **IVC CI < 20% with change in CO -10% to +10% (n=14)** | | | | | | | | | | | | | | |
| Pt 8: 0.0 ^f^ | IHD | 3.2 | C | -62.2 | -16.23 | -40.2 | -5.02 | +9.4 |  |  | 3 | N | 7 | 2,4,7,8b,d |
| Pt 9: 0.0 | IHD^a^ | 4.3 | B | -29.0 | -8.29 | -23.9 | -3.99 | -6.5 | Norepi 0.02 | 0 | 4 ^l^ | Y | 12 | 2,4 |
| Pt 2: 2.2 | IHD | 1.0 | B | -27.6 | -7.90 | -22.4 | -2.80 | +6.0 | Dopa 11 | ↑ | 2b | Y | 11 | 2,4,6,8c |
| Pt 1: 11.9^d^ | IHD^a^ | 2.5 | B | -29.0 | -12.87 | -21.6^n^ | -4.32 | -6.2 | Epi 0.06  Vaso 0.9 | ↑↑↑  0 | 3 | Y | 13 | 1,4 |
| Pt 10: 0.0 | IHD | 4.9 | B | -24.1 | -6.89 | -16.1^i^ | -4.03 | 0.0 |  |  | 2a | Y | 10 | 2,4,8b,d |
| Pt 1: 2.3 | CRRT | 1.1 | B |  |  | -14.0 | -3.49 | +0.51 | Norepi 0.07 | 0 | 2b | Y | 15 | 1,4,8a,c |
| Pt 1: 2.1 | CRRT | 1.1 | B |  |  | -13.0 | -3.25 | -2.0 | Norepi 0.08 | 0 | 2b | Y | 15 | 1,4,8a,c |
| Pt 11: 0.7 | IHD | 10.0 | B | -2.3 | -5.55 | -12.8 | -0.42 | -3.7 | Norepi 0.03 | 0 | 2b | N | 13 | 1,4,8f |
| Pt 1: 12.8^d^ | CRRT | 2.4 | A |  |  | -5.3 | -2.66 | -2.1 | Epi 0.02  Vaso 0.44 | ↑↑↑  0 | 2b | Y | 14 | 1,4 |
| Pt 11: 0.0 | IHD | 2.0 | A | -7.0 | -2.34 | -4.0 | -0.30 | +4.3 | Norepi 0.013 | 0 | 2b | Y | 13 | 1,4,8f |
| Pt 7: 0.0 | CRRT | 3.0 | A |  |  | -3.8 | -0.63 | +7.6 | Dobuta 4  Epi 0.05 | ↑↑  ↑↑↑ | 2b | Y | 14 | 2,4,8d |
| Pt 1: 13.1^d^ | CRRT | 2.4 | A |  |  | -1.1^n^ | -0.28 | +5.2 | Epi 0.01  Vaso 0.44 | ↑↑↑  0 | 2b | Y | 14 | 1,4 |
| Pt 1: 12.1^d,g^ | CRRT | 2.5 | A |  |  | -1.5 | -0.36 | +2.5 | Epi 0.05  Vaso 0.89 | ↑↑↑  0 | 2b | Y | 17 | 1,4 |
| Pt 1: 10.0 | CRRT | 13.3 | A |  |  | +23.6**^k^** | +5.89 | -7.7 | Epi 0.08 | ↑↑↑ | 2b | Y | 16 | 1,4 |
| **IVC CI < 20% with decrease in CO < -10% (n=13)** | | | | | | | | | | | | | | |
| Pt 1: 3.5 | IHD^a^ | 0.0 | C | -50.7 | -14.49 | -43.4 | -10.85 | -22.4 | Norepi 0.05 | 0 | 2b | Y | 15 | 1,4,8a,c |
| Pt 1: 8.0 | IHD | 7.3 | C | -53.3 | -13.1 | -41.7 | -5.22 | -14.7 |  |  | 3 | Y | 9 | 1,4,8a,c |
| Pt 12: 0.0 | CRRT | 10.4 | C |  |  | -37.5 | -1.56 | -23.0 | Norepi 0.10  Vaso 0.96 | 0  0 | 3 | N | 15 | 1,4 |
| Pt 9: 1.7 | IHD | 3.1 | B | -28.5 | -6.99 | -15.4 | -1.63 | -56.5 | Norepi 0.10  Vaso 0.95 | 0  0 | 3 | Y | 16 | 2,4 |
| Pt 13: 1.1 | IHD^a^ | 15.8 | B | -17.0 | -5.68 | -13.2 | -1.06 | -20.0 | Norepi 0.01 | 0 | 3 | Y | 15 | 2,5,6,8c |
| Pt 1: 2.0 | CRRT | 1.1 | B |  |  | -9.4 | -2.34 | -22.3 | Norepi 0.09 | 0 | 2b | Y | 15 | 1,4,8a,c |
| Pt 1: 8.3 | IHD | 7.3 | B | -13.3 | -3.71 | -7.4 | -1.85 | -13.5 |  |  | 3 | N | 14 | 1,4,8a,c |
| Pt 1: 12.3^d^ | CRRT | 2.5 | A |  |  | -4.8^n^ | -1.20 | -15.7 | Epi 0.05  Vaso 0.89 | ↑↑↑  0 | 2b | Y | 14 | 1,4 |
| Pt 1: 13.3^d^ | CRRT | 2.4 | A |  |  | -5.5 | -1.39 | -13.2 | Epi 0.01  Vaso 0.44 | ↑↑↑  0 | 2b | Y | 14 | 1,4 |
| Pt 1: 10.3 | CRRT | 13.3 | A |  |  | -4.3 | -1.08 | -18.1 | Epi 0.09 | ↑↑↑ | 3 | Y | 14 | 1,4 |
| Pt 14: 0.0 | IHD | 0.0 | A | -1.2 | -0.59 | -1.2 | -0.30 | -27.4 |  |  | 4^m^ | N | 4 | 2,4,6,8c |
| Pt 1: 0.0 | IHD | 0.0 | A | +2.8 | +2.84 | +3.9 | +1.02 | -19.4 | Dopa 7  Norepi 0.14  Phen 0.28 | ↑↑  0  ↓ | 4 ^m^ | Y | 18 | 1,4,8a,c |
| Pt 15: 0.0 | IHD | 0.0 | A | +3.3 | +4.45 | +8.8 | +0.68 | -13.5 | Phen 0.55  Norepi 0.04 | ↓  0 | 4 ^m^ | Y | 17 | 1,4,8c |
| **NOT RELATIVE INTRAVASCULAR OVERLOADED**  **IVC CI > 20% with increase in CO > 10% (n=1)** | | | | | | | | | | | | | | |
| Pt 16: 0.0 | IHD | 34.1 | D | +7.0 | +3.49 | +14.9 | +3.72 | +12.6 | Dopa 8 | ↑↑ | 2b | Y | 19 | 1,4,8a |
| **IVC CI > 20% with change in CO -10% to+10% (n=8)** | | | | | | | | | | | | | | |
| Pt 17: 0.8 | IHD | 60.9 | E | -36.4 | -10.41 | -26.5 | -3.3 | +8.1 | Epi 0.14 | ↑↑↑ | 2a | Y | 19 | 1,4,5,8b |
| Pt 18: 2.5 | IHD | 47.0 | E | -30.3 | -10.12 | -22.4 | -2.49 | +2.4 |  |  | 3 | Y | 10 | 3,4 |
| Pt 6: 1.2 | IHD | 37.4 | E | -17.2 | -4.30 | -14.9 | -3.43 | -3.4 | Norepi 0.04  Dopa 7 | 0  ↑↑ | 2b | Y | 11 | 2,4,7,8c,d |
| Pt 19: 0.0 | CRRT | 37.7 | D |  |  | -3.5 | -0.28 | +1.5 | Dopa 5 | ↑↑ | 4 ^h^ | N | 11 | 1,5,7,8 |
| Pt 6: 4.8 | IHD | 35.8 | D | -7.4 | -1.86 | +0.**7^i^** | +0.09 | +5.3 | Norepi 0.11  Dopa 12  Dobut 2.7 | 0  ↑  ↑↑ | 2b | Y | 15 | 2,4,7,8c,d |
| Pt 20: 0.0 | IHD | 33.3 | D | -1.2 | -0.79 | +2.9 | +0.37 | +2.3 | Norepi 0.03 | 0 | 2b | Y | 18 | 2,5,7 |
| Pt 2: 8.9 | IHD^a^ | 32.7 | D | +3.6 | +6.15 | +3.2 | +1.07 | 2.9 | Dopa 9 | ↑↑ | 4 ^m^ | N | 12 | 2,4,6,8 |
| Pt 13: 0.0 | CRRT | 23.4 | D |  |  | +5.0**^k^** | +0.36 | -7.7 | Norepi 0.03 | 0 | 2b | Y | 16 | 2,5,6,8a,c |
| **IVC CI > 20% with decrease in CO <-10% (n=7)** | | | | | | | | | | | | | | |
| Pt 1: 4.3 | IHD^a^ | 22.2 | F | -50.5 | -12.37 | -40.2 | -5.13 | -11.4 | Norepi 0.03 | 0 | 3 | Y | 14 | 1,4,8a,c |
| Pt 21: 0.0 | IHD | 20.4 | E | -29.9 | -7.47 | -24.9 | -6.23 | -15.2 | Dopa 3 | ↑↑ | 3 | Y | 10 | 2,4,5,7,8b |
| Pt 22: 0.0 | IHD | 21.1 | E | -22.9 | -8.80 | -18.2 | -2.14 | -15.6 |  | 0 | 4 | N | 7 | 3,4,8 |
| Pt 17: 0.0 | IHD | 37.7 | E | -22.9 | -6.53 | -9.2**^j^** | -1.02 | -28.3 | Epi 0.04 | ↑↑↑ | 2b | N | 18 | 1,4,5,8b |
| Pt 18: 0.0 | IHD | 34.1 | E | -28.5 | -7.14 | -7.1**^i^** | -0.34 | -44.4 | Norepi 0.04 | 0 | 2b | Y | 15 | 3,4 |
| Pt 6: 0.0 | IHD | 24.3 | D | -4.5 | -1.09 | +1.7**^k^** | +0.21 | -16.2 | Dopa 17  Norepi 0.05 | ↑  0 | 2b | N | 9 | 2,4,7,8c,d |
| Pt 16: 2.6 | CRRT | 32.5 | D |  |  | +4.6 | +0.62 | -38.3 | Dopa 22  Norepi 0.14  Vaso 0.93 | ↑  0  0 | 2b | Y | 19 | 1,4,8a |

**Notes:** Patients were de-identified as Pt Number: Encounter time (in days and fractions of days, with time 0 being the time of the first CO measurement pre-IHD or during CRRT).

Encounters were categorized first on IVC CI < 20% or > 20%, then by change in CO as > +10%, +10% to -10%, and > -10%, then ranked by net volume change between CO measurements normalized for body weight.

0.9% saline volume equivalent of pRBCs was estimated to be 2.8 * pRBC volume (See Supplemental figure 1)

Hemodialysate bath: Ca^++^ 2.5 mEq/L, Na^+^ 138 to 142 mEq/L, K^+^ 3 to 4 mEq/L, Temperature 36 to 37 degrees Celsius. HCO3^-^ 25 to 30 mEq/L for IHD and 32 mEq/L for CRRT.

^a^UF only, no HD

^b^ Categories for IDH severity: 0, no criteria for IDH; 1, 0.9% saline > 500 mL or albumin; 2a, MAP < 65 during HD and no vasopressors; 2b, constant dose of vasopressors; 3, SBP decreased > 50 mmHg or MAP decreased > 20%; 4, vasopressors started or increased and/or HD stopped < 2 h due to hypotension

^c^HFpEF criteria[^48^](#_ENREF_48)

^d^s/p mitral valve replacement 11 days after the first encounter

^e^Outliers

^f^Intra-aortic balloon pump

^g^IVC pre CRRT

^h^CRRT stopped for intractable hypotension.

pRBC volumes :^i^300-350mL, ^j^500mL, ^k^640 mL

^l^Norepinephrine dose increased for 30 minutes during HD

^m^Intermittent dialysis discontinued for intractable hypotension after < 2 hours

^n^25% albumin administered. Only 3 encounters received 100 mL of 25% albumin, while no encounters received 5% albumin or fresh frozen plasma during the cardiac output intervals. Immediate volume effect of 25% albumin is 5 times that of 0.9% saline,[^38^](#_ENREF_38) but decreases over time with a half-time of 2.5 hours.[^37^](#_ENREF_37) Thus for two CRRT encounters where 100 cc of 25% albumin were given 2.5 hours prior to the CO measurement, the albumin has a dilution effect of 100 cc and an initial expansion effect of 400 cc which decays to an expansion effect of 200 cc volume expansion at 2.5 hours with a net effect at 2.5 hours of 300 cc. Similarly for one IHD encounter where 100 cc of 25% albumin was given 3.5 hours prior to the CO measurement, the albumin has a net effect of 252 cc.

Disease states: 1= AKI, 2 = AKI/CKD, 3 = ESRD, 4 = heart failure, 5 = sepsis and/or shock, 6 = severe pulmonary hypertension, 7 = STEMI or NSTEMI, 8 = other chronic cardiac disease including CAD (n=6), atrial fibrillation/flutter (n=6), non-ischemic dilated cardiomyopathy (n=3), severe mitral valve disease (n=2), 8a = severe mitral regurgitation (MR), 8b = severe MR, 8c=moderate to severe tricuspid regurgitation, 8d=HFrEF, 8e=HFmrEF, 8f=HFpEF.

**Abbreviations:** AKI = acute kidney injury, CAD = coronary artery disease, CKD = chronic kidney disease, CRRT = continuous renal replacement therapy, Dopa = dopamine, Dobut = dobutamine, Epi=epinephrine, ESRD = end-stage renal disease, HFrEF = heart failure with reduced ejection fraction, HFmrEF = heart failure with mid-range ejection fraction, HFpEF = heart failure with preserved ejection fraction, IDH = intradialytic hypotension, IHD = intermittent hemodialysis, IVC CI = inferior vena cava collapsibility index, MR = mitral regurgitation, NSTEMI = Non-ST elevation myocardial infarction, Norepi = norepinephrine, Phen= phenylephrine, STEMI = ST elevation myocardial infarction, Vaso = vasopressin, UF = ultrafiltration only

## Supplemental Table 2: Distribution of encounters from Supplemental Table 1 and Figure 1b.

| Number of encounters based on IVC CI, net weight-adjusted volume removed, and change in cardiac output | | | | |
| --- | --- | --- | --- | --- |
|  | Volume removed < 7 mL/kg or volume added | Volume removed 7 mL/kg to 30 mL/kg | Volume removed >30 mL/kg |  |
| IVC CI <20% | Sector A | Sector B | Sector C |  |
| CO increased > 10% | 1 | 12 | 2 |  |
| CO within +/- 10% | 6 | 7 | 1 |  |
| CO decreased > 10% | 6 | 4 | 3 |  |
| IVC CI > 20% | Sector D | Sector E | Sector F |  |
| CO increased > 10% | 1 | 0 | 0 |  |
| CO within +/- 10% | 5 | 3 | 0 |  |
| CO decreased > 10% | 2 | 4 | 1 |  |
|  |  |  |  |  |
| Relationship of net weight adjusted volume change and change in CO (IVC CI <20%) (number of encounters) | | | | |
|  | Volume removed < 7 mL/kg or volume added | Volume removed 7 mL/kg to 30 mL/kg | Volume removed > 30 mL/kg |  |
|  | Sector A | Sector B | Sector C | p=0.0164 (LLR) |
| CO increased > 10% | 1↓ | 12↑ | 2 |  |
| CO not increased > 10% | 12 | 11 | 4 |  |
|  |  |  |  |  |
| Relationship of severity of IDH and net weight-adjusted volume removed (IVC CI <20%) (number of encounters) | | | | |
|  | Volume removed < 7 mL/kg or volume added | Volume removed 7 mL/kg to 30 mL/kg | Volume removed > 30 mL/kg |  |
|  | Sector A | Sector B | Sector C | p= 0.0095 (LLR) |
| IDH 2a | 0 | 3 | 0 |  |
| IDH2b | 9 | 11 | 1 |  |
| IDH3 | 1↓ | 8 | 5↑ |  |
| IDH4 | 3↑ | 1 | 0 |  |
|  |  |  |  |  |
| Relationship of severity of IDH to change in CO (number of encounters) | | | | |
|  | CO increased > 10% | CO within +/-10% | CO decreased > 10% | p = 0.047 by LLR |
| IDH 2a | 2↑ | 2 | 0↓ |  |
| IDH 2b | 8 | 14↑ | 8↓ |  |
| IDH 3 | 6 | 3↓ | 8↑ |  |
| IDH 4 | 0↓ | 3 | 4↑ |  |

Abbreviations: CO = cardiac output, IDH = intradialytic hypotension category (see Supplemental Table 1), IVC CI = inferior vena cava collapsibility index, LLR = log likelihood ratio, ↓ = number of encounters is fewer than what would be expected due to chance, ↑= number of encounters is more than what would be expected due to chance
