# Supplementary material for: Changes in cardiac output with hemodialysis relate to net volume balance and to inferior vena cava ultrasound collapsibility in critically ill patients
Source: Ren Fail. 2020 Feb 12;42(1):179–92. doi: 10.1080/0886022X.2020.1726384 (PMC7034082; doi:10.1080/0886022X.2020.1726384)
Supplement: Supplemental Material [file IRNF_A_1726384_SM8379.docx]

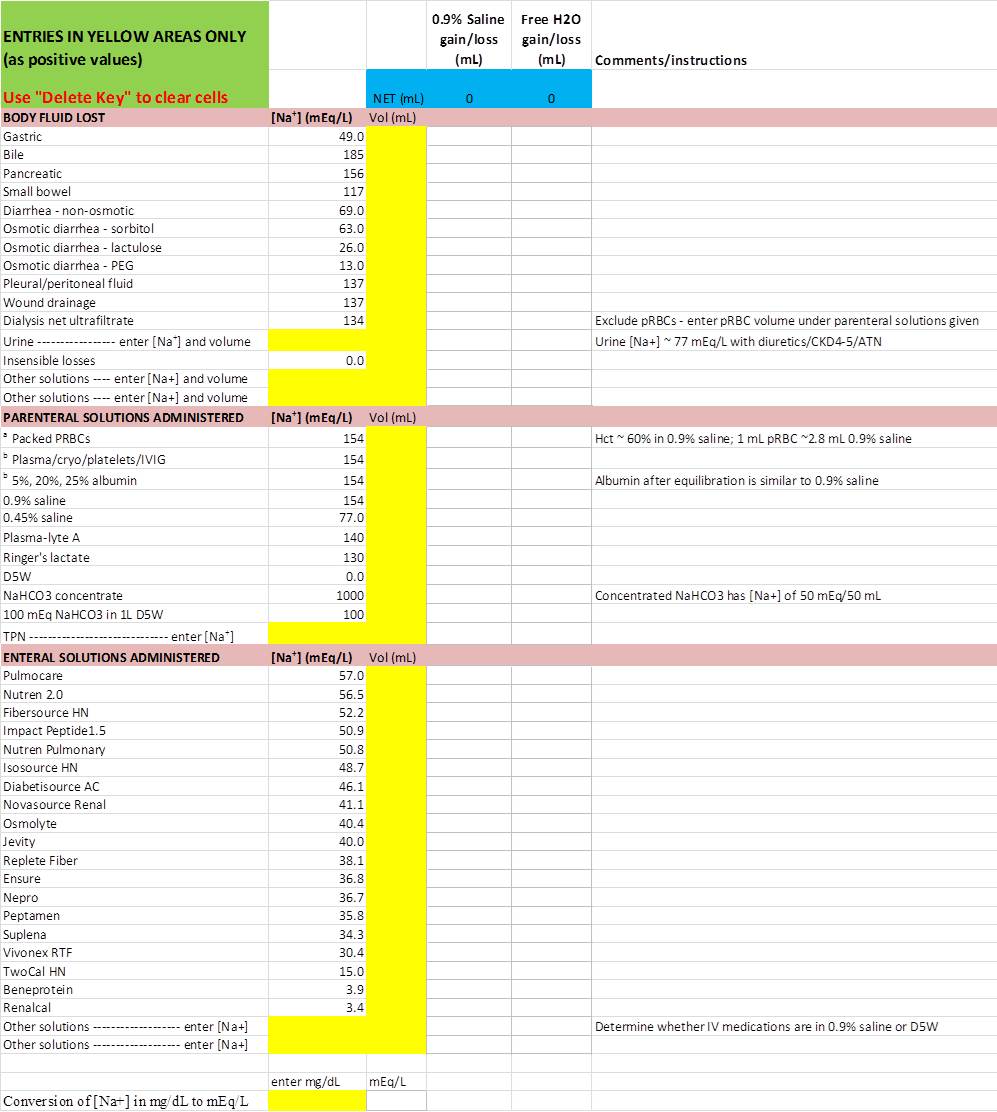


## **Legend for Supplemental Figure 1**

Calculator used to estimate isonatremic volume (0.9% saline) and water equivalents of body fluid losses and parenteral and enteral fluid inputs. [^32^](#_ENREF_32)

Volumes of body fluids lost, and of parenteral and enteral fluids given are entered into the calculator and volumes of 0.9% saline and water lost or gained are generated based on population derived sodium concentrations in various body fluids. [^32^](#_ENREF_32)

^a^Using an additive solution (AS) system, average Hct of 60% with average sodium concentration of 155 mEq/L in diluent.[^35^](#_ENREF_35) pRBC volume x 2.8 = 0.9% saline equivalent volume.[^35^](#_ENREF_35)

^b^Plasma volume effect of 5% albumin and 0.9% saline have been shown to be similar during HD.[^39^](#_ENREF_39) Plasma volume effect of fresh frozen plasma, cryoglobulin, platelets, intravenous immunogloblin and 5% albumin have been shown to be similar.[^37^](#_ENREF_37)

Shortly after IV administration, 25% albumin has an oncotic effect equivalent to a five-fold plasma volume[^38^](#_ENREF_38) and the effective plasma expansion has a half-time of 2.5 hours.[^37^](#_ENREF_37) UF and net volumes were manually corrected for the time interval between administration and CO measurement for the 100 cc of 25% albumin given for individual encounter data in Supplemental Table 1. Albumin as 5%, 20% or 25% has a similar volume effect after equilibration (5 half times, ~12.5 hours).
